# Hypoxic bone marrow mesenchymal cell‐extracellular vesicles containing miR‐328‐3p promote lung cancer progression via the NF2‐mediated Hippo axis

**DOI:** 10.1111/jcmm.15865

**Published:** 2020-11-21

**Authors:** Xi Liu, Feng Jiang, Zhilinag Wang, Lang Tang, Bin Zou, Pengfei Xu, Tenghua Yu

**Affiliations:** ^1^ Department of Thoracic Surgery JiangxiCancer Hospital Nanchang China; ^2^ Department of Obstetrics and Gynecology The Second Affiliated Hospital of Chongqing Medical University Chongqing China; ^3^ Department of Breast Surgery JiangxiCancer Hospital Nanchang China

**Keywords:** bone marrow mesenchymal cells, extracellular vesicles, hippo pathway, hypoxic, lung cancer, microRNA‐328‐3p, NF2

## Abstract

Lung cancer is the most aggressive tumour afflicting patients on a global scale. Extracellular vesicle (EV)‐delivered microRNAs (miRs) have been reported to play critical roles in cancer development. The current study aimed to investigate the role of hypoxic bone marrow mesenchymal cell (BMSC)‐derived EVs containing miR‐328‐3p in lung cancer. miR‐328‐3p expression was determined in a set of lung cancer tissues by RT‐qPCR. BMSCs were infected with lentivirus‐mediated miR‐328‐3p knock‐down and then cultured in normoxic or hypoxic conditions, followed by isolation of EVs. Following ectopic expression and depletion experiments in lung cancer cells, the biological functions of miR‐328‐3p were analysed using CCK‐8 assay, flow cytometry and Transwell assay. Xenograft in nude mice was performed to test the in vivo effects of miR‐328‐3p delivered by hypoxic BMSC‐derived EVs on tumour growth of lung cancer. Finally, the expression of circulating miR‐328‐3p was detected in the serum of lung cancer patients. miR‐328‐3p was highly expressed in EVs derived from hypoxic BMSCs. miR‐328‐3p was delivered to lung cancer cells by hypoxic BMSC‐derived EVs, thereby promoting lung cancer cell proliferation, invasion, migration and epithelial‐mesenchymal transition. miR‐328‐3p targeted NF2 to inactivate the Hippo pathway. Moreover, EV‐delivered miR‐328‐3p increased tumour growth in vivo. Additionally, circulating miR‐328‐3p was bioactive in the serum of lung cancer patients. Taken together, our results demonstrated that hypoxic BMSC‐derived EVs could deliver miR‐328‐3p to lung cancer cells and that miR‐328‐3p targets the NF2 gene, thereby inhibiting the Hippo pathway to ultimately promote the occurrence and progression of lung cancer.

## 1 INTRODUCTION

Lung cancer is the most prevalent malignancy across the world, accounting for the highest number of cancer‐related deaths, with a poor 5‐year overall survival rate of only 17%.^1^ Especially poor prognosis is associated with the distant metastases already present in the majority of lung cancer patients at their first diagnosis.^2^ Smoking, environmental and occupational factors are well‐known as the leading contributors for the occurrence of lung cancer.^3^ Unfortunately, patients with lung cancer usually suffer from local or metastatic pain in addition to a great deal of emotional distress, which can seriously hamper their quality of life.^4^, ^5^ Currently, major treatment regimens for lung cancer include the use of radiation therapy and chemotherapy.^6^, ^7^ However, it is noteworthy that extracellular vesicles (EVs) have been reported as potential biomarkers for the early diagnosis of lung cancer.^8^

Extracellular vesicles are signalling organelles released by a variety of cell types, which are generally classified as exosomes, shedding microvesicles and apoptotic blebs.^9^ EVs are regarded as potential tools for cell‐cell communication and possess the ability to carry and deliver as cargo various bioactive molecules, such as microRNAs (miRs), messenger RNAs (mRNAs) and proteins.^10^ Meanwhile, bone marrow‐derived mesenchymal stem cells (BMSCs) are believed to be a part of the tumour microenvironment,^11^ whereas hypoxic conditions can regulate the biological functions of the MSCs.^12^ Intriguingly, a recent study reported that several miRs, including miR‐328‐3p, were highly expressed in exosomes derived from hypoxic BMSCs.^13^ In addition, another study reported that miR‐328 serves as a promising diagnostic biomarker for the diagnosis of early‐stage non‐small cell lung cancer.^14^ Moreover, inhibition of miR‐328 was reported to likely present a novel target for the treatment of lung cancer.^15^ Therefore, we selected miR‐328‐3p as the research focus of the current study, and prediction results revealed neurofibromin 2 (NF2) as its target gene. As the upstream gene of the Hippo pathway, NF2 is considered to be a tumour suppressor in multiple human tumours.^16^ Inactivation of NF2 was found to be responsible for refractoriness to erlotinib treatment and progression of lung cancer cells.^17^, ^18^ Additionally, several reports have highlighted that an inhibited Hippo pathway promotes the proliferation, invasion and migration of lung cancer cells.^19^, ^20^ Considering the aforementioned findings and reports, we tested the hypothesis that hypoxic BMSC‐derived EVs containing miR‐328‐3p affects the occurrence and progression of lung cancer via the Hippo pathway through targeting of NF2 gene.

## 2 MATERIAL AND METHODS

### 2.1 Ethics statement

The current study was conducted with the approval of the Ethics Committee of Jiangxi Cancer Hospital. Signed informed consents were obtained from all participating patients prior to the study, and all protocols were conducted in line with the ethics statement of the *Declaration of Helsinki*. All animal experiments were performed in strict accordance with the Guide for the Care and Use of Laboratory Animals of the National Institutes of Health, and extensive efforts were made to minimize the number and discomfort of the included animals.

### 2.2 Bioinformatics analysis

The miR data set, GSE119790, for BMSC‐derived EVs in lung cancer was retrieved from the Gene Expression Omnibus (GEO) database (https://www.ncbi.nlm.nih.gov/geo/). The data set comprised of two samples, including one normoxic sample as control and one hypoxic sample.

### 2.3 Study subjects

A total of 52 pairs of cancer tissues and adjacent normal tissues (more than 5 cm away from cancer tissues) were collected from 52 lung cancer patients (39 men and 16 women, aged 39‐80 years) from 2017 to May 2019. Based on the classification standards issued by World Health Organization for Lung Cancer in 2015, the included population had 4 patients with adenocarcinoma, 10 patients with squamous cell carcinoma, and the remaining with NSCLC. Based on the tumour‐node‐metastasis (TNM) staging system, there were 19 cases at Stages I‐II and 33 cases at Stage IIIa. The inclusion criteria were as follows: (a) patients had no malignant tumour; (b) patients did not receive any treatment, such as chemotherapy, radiotherapy or other treatments prior to the surgery described in this study; and (c) all collected samples were checked and confirmed by pathologists. In addition, lung cancer patients and 30 middle‐aged healthy volunteers (control group) donated blood samples for further analysis.

### 2.4 Cell treatment

BMSCs purchased from the American Type Culture Collection (ATCC) were cultured in the mesenchymal stem cells conditioned medium (HUXMA‐03011‐440, Cyagen Biosciences Inc) with 90% humidity and 5% CO_2_ in air. Subsequently, hypoxia induction was performed. In brief, cells were cultured with 20% oxygen (normoxic) or 1% oxygen (hypoxic) in nitrogen in a 3 gas incubator (Binder).^13^, ^21^ To inhibit the expression of miR‐328‐3p, the oligonucleotide of miR‐328‐3p was cloned into the lentiviral vector prenti‐u6‐pgkpuro, followed by infection of BMSCs.

Lung cancer cell lines A549 and H125 purchased from ATCC^13^ were cultured in Roswell Park Memorial Institute (RPMI)‐1640 medium (sh30809.01b, HyClone Company) with 90% humidity and 5% CO_2_ in air. These cell lines were then cultured under normoxic conditions (20%). Next, EVs were isolated from 5 × 10^9^ BMSCs under normoxia or anoxia, and A549 and H125 cells were seeded into a 6‐well plate a day prior to treatment. When the A549 and H125 cells reached about 70% confluence, 20 µg/mL EVs were directly added to the cells, with the addition of phosphate buffered saline (PBS) as control. After 48 hours of treatment, cells were collected for subsequent experimentation.

### 2.5 Isolation of EVs

Foetal bovine serum (FBS) was centrifuged at 100 000 *g* and 4°C for 18 hours to remove EVs, and the supernatant of FBS was collected and filtered through a 0.22 µm filter (Millipore). BMSCs were cultured in a medium supplemented with FBS (EVs were removed). After 72 hours, the cell culture medium was collected and centrifuged at 300 *g* for 10 minutes, at 2000 *g* for 15 minutes and at 12 000 *g* for 30 minutes to remove the floating cells and cell fragments. Next, the obtained supernatant was passed through a 0.22 µm filter (Millipore), followed by resuspension with PBS and centrifugation under the same conditions mentioned above. All ultrafiltration steps were conducted at 4°C in a Beckman ultracentrifuge (Optima L‐90K) using a SW‐32Ti rotor. The precipitation of EVs was quantified by bicinchoninic acid (BCA) protein analysis (Sigma) and then resuspended in 100 µL sterile PBS. Five patients and 5 healthy controls were randomly selected, from whom plasma EVs were isolated. In brief, the blood was collected into an EDTA‐K2 anticoagulant tube and immediately mixed to avoid coagulation. The method for isolation of EVs was the same as mentioned above. The isolated EVs were either immediately used or stored at −80°C for subsequent analysis.^13^

### 2.6 Electron microscopy

The isolated 20 µg samples of EVs were dropped onto a carbon‐coated electron microscope grid and fixed with 0.1 mol/L sodium carbonate buffer (pH value of 7.3) containing 2% glutaraldehyde and 2% polyformaldehyde at room temperature for 3 hours. The samples were dried at the critical point and then installed on the sample post, and the coating was sputtered. Finally, the samples were observed under a Hitachi S3400 scanning electron microscope.^21^

### 2.7 Nanoparticle tracking analysis (NTA)

The size distribution and concentration of EVs were determined by NTA following the manufacturer's instructions. The properties of light scattering and Brownian motion (Zetasizer Nano zs90 instrument) were recorded in this analysis. The EVs were resuspended and mixed in 1 mL PBS, and the diluted EVs were then injected into a Zetasizer Nano ZS90 instrument. Next, the particles were tracked according to Brownian motion and diffusion coefficient, and the particle sizes were measured. The filtered PBS was used as the control. NP100 films were employed for all samples, and the measurement parameters were set to 44.5 mm and 0.64 V. Calibration samples were diluted with CPC100 standard particles (dilution ratio of 1:1000) at the same setting. Five videos were recorded, usually lasting 60 seconds. The data were analysed and optimized using Zetasizer software to identify and track each particle frame by frame.^13^

### 2.8 Treatment of EVs

GW4869 (Sigma‐Aldrich Chemical Company) is wildly used as an inhibitor of exogenesis/release. In this experiment, GW4869 was added to the culture medium containing 10% EV‐free FBS before BMSCs were placed in the anoxic chamber. Three days after hypoxic treatment, BMSC conditioned medium was collected for EV isolation, as previously described.^13^

We subsequently conducted endocytosis of EVs. Specifically, EVs labelled with green fluorescent lipophilic dye PKH67 were isolated from the culture medium in accordance with the manufacturer's instructions (Sigma‐Aldrich Chemical Company). Then, 20 µg EVs were mixed with 4 µL PKH67 in 1 mL diluent C and cultured at room temperature for 4 minutes. The same amount of EV‐free FBS was added to stop the reaction before the occurrence of a rejection. EVs were rinsed twice with FBS/RPMI‐1640 to remove the excess PKH67. The labelled EVs were then resuspended in 1 × PBS and used immediately or stored at −20°C. The prepared fluorescence‐labelled EVs were resuspended and added to A549 or H125 cells with 80% confluence, followed by 5‐hour incubation. Cells were then rinsed twice with PBS and fixed with 4% paraformaldehyde at room temperature for 30 minutes. Following another 3 rinses with PBS, the cells were stained with a medium containing 4′,6‐diamidino‐2‐phenylindole (DAPI) (VectorShield) for 5 minutes. After staining, cells were rinsed twice with PBS to remove the excess DAPI, sealed and observed under a fluorescence microscope (BX53, Olympus).^13^

### 2.9 Reverse transcription‐quantitative polymerase chain reaction (RT‐qPCR)

Cellular or extracellular RNA content was extracted utilizing a TRIzol kit (TR118, MRC). A total of 25 fmol *C elegans* cel‐miR‐39 (Ribobio) was added to each sample before the extracellular RNA (EVs) was isolated. A RevertAid First Strand cDNA Synthesis Kit (Thermo Fisher Scientific Inc) was applied to reverse‐transcribe the total RNA into complementary DNA (cDNA). miRNA expression was quantified using a NCode EXPRESS SYBR GreenER miRNA RT‐qPCR kit (Invitrogen) with an ABI PRISM 7300 Sequence Detection System (Applied Biosystems). The expression of NF2 was determined using PrimeScript RT‐PCR kits (Takara). Β‐actin was used as the internal reference for the NF2 gene, cel‐miR‐39 for quantitative expression of miRNA in EVs or supernatant and U6 for quantitative expression of miRNA in cells.^21^ All the primers were purchased from Ribobio, as shown in Table 1. Fold changes in gene expression were calculated by means of the relative quantification (2^−ΔΔCt^ method). Three duplicates were set for each sample.^22^

**Table 1:** Primer sequences for RT‐qPCR

Target gene	Primer sequence (5′‐3′)
miR‐328‐3p	F: CGGGCCTGGCCCTCTCTGCC
RNU6	F: CTCGCTTCGGCAGCACA
cel‐miR‐39	F: GGTCACCGGGTGTAAATCAGCTTG
NF2	F: TTGCGAGATGAAGTGGAAAGG
	R: CAAGAAGTGAAAGGTGACTGGTT
β‐actin	F: TCACCCACACTGTGCCCATCTACGA
	R: CAGCGGAACCGCTCATTGCCAATGG

Abbreviations: F, forward; miR−, microRNA−; NF2, neurofibromatosis 2; R, reverse; RNU6, U6; RT‐qPCR, reverse transcription‐quantitative polymerase chain reaction.

### 2.10 Western blot analysis

Protein content was extracted with radioimmunoprecipitation assay buffer containing protease inhibitors (Roche Diagnostics GmbH). A BCA™ protein detection kit (Pierce) was applied for protein quantification. Next, 30 mg protein was separated on 10% sodium dodecyl sulphate‐polyacrylamide gel electrophoresis gel and transferred to a polyvinylidene fluoride membrane (Millipore). The membrane was then sealed with 5% blocking buffer and incubated overnight with the following rabbit antibodies to HSP70 (dilution ratio of 1:1000, ab181606), glyceraldehyde‐3‐phosphate dehydrogenase (GAPDH; dilution ratio of 1:10 000, ab8245), calnexin (dilution ratio of 1:1000, ab10286), histone (dilution ratio of 1:1000, ab1791), CD63 (dilution ratio of 1:5000, ab134045), Tumour Susceptibility Gene 101 (TSG101, ab30871), N‐Cadherin (dilution ratio of 1:1000, ab18203), E‐Cadherin (dilution ratio of 1:50, ab1416), Vimentin (dilution ratio of 1:1000, ab137321), NF2 (dilution ratio of 1:50, ab88957), PDZ‐binding motif (TAZ; dilution ratio of 1:500, ab224239), yes‐associated protein 1 (YAP1; dilution ratio of 1:5000, ab52771), phosphorylated (p)‐YAP1 (dilution ratio of 1:10 000, ab76252) and large tumour suppressor gene 1 (LATS1; Cat#3477), p‐LATS1 (Cat#8654). Horseradish peroxidase conjugated anti‐mouse or anti‐rabbit against immunoglobulin G (IgG) was used as the secondary antibody (diluted in Tween‐20 at a ratio of 1:5000). All the aforementioned antibodies were purchased from Abcam Inc, except for LATS1 and p‐LATS1, which were purchased from Cell Signaling Technology. The cytoplasmic and nuclear extracts in the lung cancer cells were separated using a Nuclear and Cytoplasmic Protein Extraction Kit (Beyotime) following the manufacturer's instructions. A densitometer (GS‐700; Bio‐Rad Laboratories) was applied to scan the bands, followed by quantification conducted using the Quantity One 4.4.0 software.^21^, ^23^ The experiment was repeated in triplicate with independent samples.

### 2.11 Analysis of cell biology

Cell proliferation was assayed using a cell counting kit‐8 (CCK‐8) (Dojindo) following the manufacturer's instructions. A549 or H125 cells were seeded together with 20 µg/mL different EVs in 96‐well plates. Under the standard conditions of cell culture, cells were incubated with CCK‐8 solution for 1 hour, and then, the fraction of living cells was determined at the optimal optical density of 450 nm using a microplate reader (Multiskan Sky Microplate Spectrophotometer, Cat. No. 51119570, Thermo Fisher Scientific Inc). In addition, A549 or H125 cells were treated with different EVs for 24 hours. Flow cytometry was applied to detect the positive rate of Annexin V‐fluorescein isothiocyanate (FITC)/propidium iodide (PI) expression on the surface of A549 or H125 cells, with analysis of cell apoptosis rate.

Cell migration and invasion were subsequently detected. In brief, the Transwell migration assay was conducted using a transit insert containing polycarbonate filters in 8‐µm wells (cat. No. 3422; Corning Glass Works). A total of 5 × 10^4^ cells were suspended in 200 µL serum‐free medium and added to the apical chamber. EVs suspended in complete medium were added to the basolateral chamber. After 24 hours, the cells were fixed with 4% paraformaldehyde, stained with crystal violet and observed under a microscope (CKX41, Olympus). Migrating cells under the membrane surface were quantified by counting in 10 randomly selected fields of view. Invasion ability of cells was detected by Matrigel matrix gel invasion assay with the use of a Transwell system (BD Biosciences). Except that the apical chamber filter (well diameter of 8 µm) was coated with 1 mg/mL matrix glue, the other operations were the same as those used in the migration experiment.

### 2.12 Dual‐luciferase reporter gene assay

The NF2 3′‐untranslated region (3′‐UTR) sequence containing mutant type (MUT) or wild‐type (WT) miR‐328‐3p‐binding site was constructed by GenScript and cloned into the pGL‐3 luciferase reporter vector. HEK293T cells were then cultured in a 24‐well plate for 24 hours. Next, the pGL‐3‐WT‐NF2 or pGL‐3‐MUT‐NF2 3′‐UTR reporter plasmids were co‐transfected with miR‐328‐3p mimic or mimic negative control (NC) with the use of Lipofectamine 3000 (Invitrogen). The activities of firefly and Renilla luciferase were then determined using a dual‐luciferase detection system (Promega). The relative expression of firefly luciferase activity was normalized to Renilla luciferase activity.^24^

### 2.13 Immunofluorescence and immunohistochemistry

After adhering to the slides, the cells were rinsed thrice with PBS for 3 minutes each time and then fixed with 4% paraformaldehyde for 15 minutes. Next, 0.5% Triton X‐100 (prepared with PBS) was applied to clear the cells at room temperature for 20 minutes. After another PBS rinse, normal goat serum was dripped on the slides to seal the cells at room temperature for 30 minutes. Enough amounts of YAP1 (dilution ratio of 1:20; ab52771) and TAZ (dilution ratio of 1:1000; ab224239) were then added to each slide, followed by overnight incubation at 4°C. The secondary antibody against IgG (dilution ratio of 1:500; ab150075) coupled with Alexa Fluor 647 was added for further 1‐hour incubation at 37°C. Finally, DAPI was used to stain the nuclei for 5 minutes in the dark. All the aforementioned antibodies were purchased from Abcam.

### 2.14 Xenograft in nude mice

A nude mouse tumour model was established by injecting 1 × 10^6^ A549 cells into BALB/c nude mice via the tail vein. In short, 50 nude mice with approximately equal bodyweights and age of 4‐6 weeks were randomly assigned into 5 groups, with 10 mice in each group. EVs were then resuspended in 100 µL sterile PBS and injected intravenously into the mice once every 2 days until tumours grew to an average 5 mm^3^ volume (100 µg each time) after A549 cell injection. The tumour volume of each mouse was measured using Vernier calipers. The mice were euthanized 30 days after A549 cell transplantation, and the tumours were dissected and weighed. Finally, HE staining was performed to stain the paraffin sections for detection of lung metastasis.

### 2.15 Statistical analysis

Data analyses were performed using the SPSS 21.0 software (IBM Corp.). Measurement data were expressed as mean ± standard deviation. Independent sample *t* test was applied to compare the unpaired data between two groups obeying normal distribution and homogeneity of variance. Data among multiple groups were compared using one‐way analysis of variance (ANOVA), with Tukey's *post hoc* test. Data among multiple groups at different time‐points were compared by repeated measures ANOVA, followed by Bonferroni *post hoc* test. Pearson's correlation analysis was performed for correlation analysis. A value of *P* < .05 was indicative of a statistical significance.

## 3 RESULTS

### 3.1 miR‐328‐3p was up‐regulated in hypoxic BMSC‐derived EVs

**FIGURE 1:**
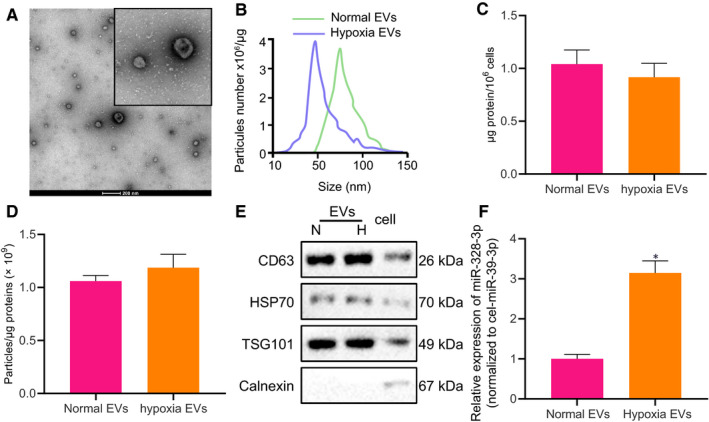
miR‐328‐3p is up‐regulated in hypoxic BMSC‐derived EVs. A, Morphological characteristics of EVs isolated from BMSCs observed under a scanning electron microscopy (scale bar = 200 nm). B, Nanosight particle tracking analysis of the EVs isolated from BMSCs cultured under normoxic and hypoxic conditions. C, Quantification of equivalent protein EVs produced by 10^6^MSCs by BCA analysis. D, Correlation of the quantity of EVs with the amount of protein. E, The presence of CD63, TSG101 and HSP70 and the absence of calnexin in BMSC‐derived EVs, as detected by Western blot analysis, normalized to GAPDH. F, Relative expression of miR‐328‐3p determined by RT‐qPCR in EVs isolated from BMSCs cultured under normoxic and hypoxic conditions, normalized to cel‐miR‐39, showing the increase of miR‐328‐3p in EVs from BMSCs cultured under hypoxic conditions. In this examination, the hypoxic extracellular vesicles were isolated from the supernatant of BMSCs cultured under hypoxic conditions, while normal extracellular vesicles were isolated from the supernatant of BMSCs cultured under normal conditions. ‘EVs’ is the abbreviation of extracellular vesicles. The experiment was repeated three times with independent samples. *P < .05 vs the EVs from BMSCs cultured under normoxic conditions. The data were measurement data and presented as mean ± standard deviation. Data between two groups were compared using independent sample*t*test

BMSC‐derived EVs were initially isolated from the supernatant of BMSCs cultured under normoxic (20%) and hypoxic (1%) conditions. The concentration and particle size of the purified EVs were examined by scanning electron microscopy and NTA; results demonstrated that the isolated EVs were typical round particles with a diameter of 50‐200 nm in the form of three‐dimensional saucer‐like structures with a clear membrane (Figure 1A, B). Analysis using BCA revealed that the preparation of EVs was normalized to 1.04 ± 0.13 µg protein/10^6^ MSCs, with no difference between hypoxia‐ and normoxia‐treated MSCs (Figure 1C). EV concentration was 1‐2 × 10^9^ EVs/µg of protein under normoxia and hypoxia culturing conditions (Figure 1D). Western blot analysis further confirmed that the isolated EVs contained the known EV marker proteins CD63, TSG101 and HSP70, but did not contain calnexin (Figure 1E).

Aiming to investigate the effects of EV‐delivered miR‐328‐3p on lung cancer, we isolated BMSC‐derived EVs for further experimentation. BMSCs were separately cultured with 20% oxygen and 1% oxygen for 3 days, and then, the EVs were extracted from the supernatant and analysed using RT‐qPCR. The results showed that miR‐328‐3p expression was up‐regulated in EVs derived from same concentration of hypoxic BMSCs (Figure 1F). These findings supported the involvement of hypoxic BMSC‐derived EVs containing miR‐328‐3p in lung cancer.

### 3.2 Hypoxic BMSC‐derived EVs containing miR‐328‐3p induced the proliferation, migration, invasion and epithelial‐mesenchymal transition (EMT) of lung cancer cells

**FIGURE 2:**
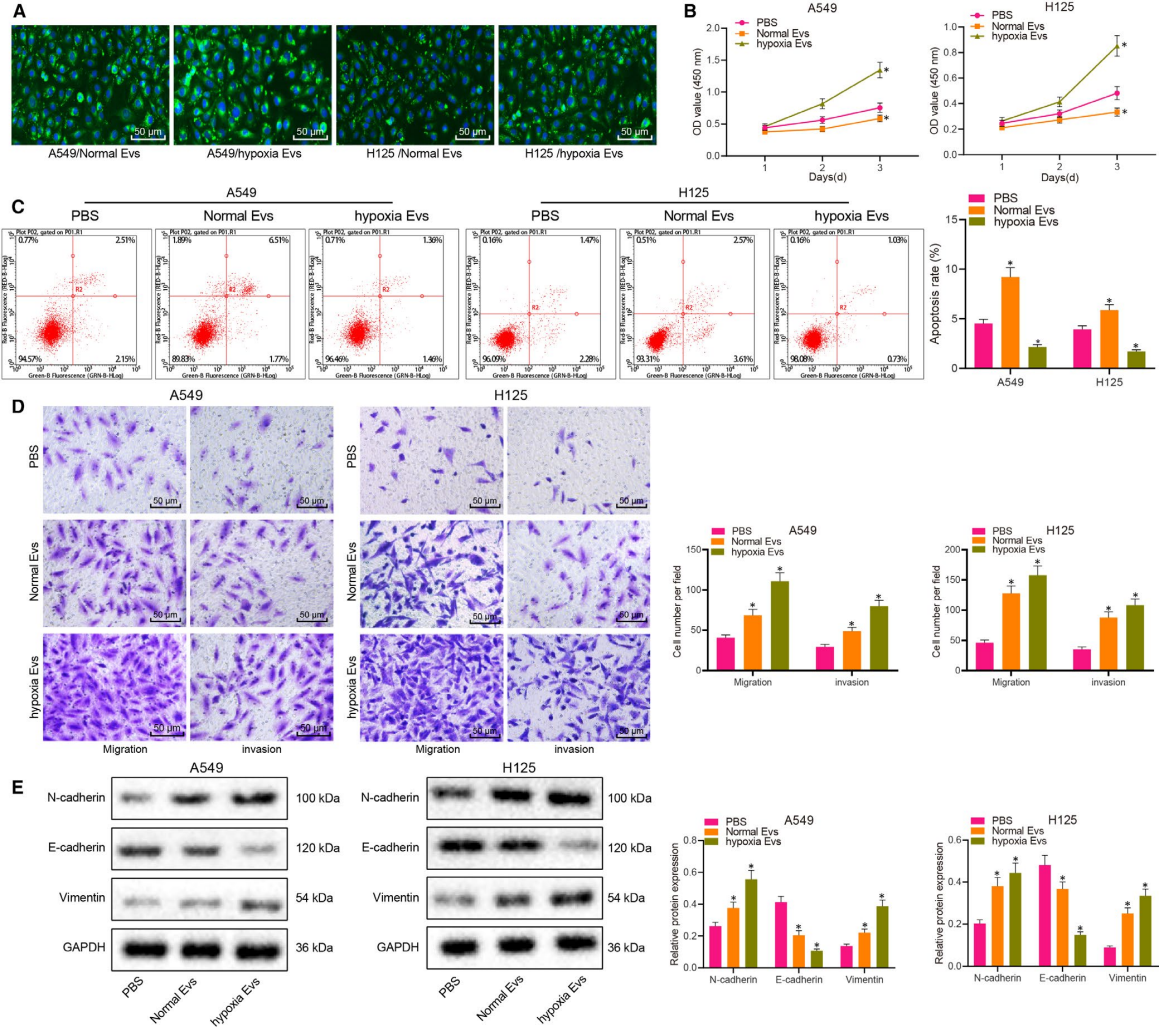
Hypoxic BMSC‐derived EVs induce the proliferation, migration, invasion and EMT of lung cancer cells in vitro. A, Uptake efficiency of PKH67‐labelled normoxic EVs or hypoxic EVs (20 µg/mL) by A549 and H12 cells as observed under a laser scanning confocal microscope (scale bar = 50 µm). B, The number of living cells at different time‐points as evaluated by CCK‐8 assay upon treatment with 20 µg/mL normoxic EVs or hypoxic EVs. C, The apoptosis of A549 and H125 cells treated with 20 µg/mL normoxic EVs or hypoxic EVs as detected by flow cytometry. D, The migration and invasion of A549 and H125 cells treated with 20 µg/mL normoxic EVs or hypoxic EVs as detected by Transwell assay (scale bar = 50 µm). E, The content of the mesenchymal markers Vimentin, N‐cadherin and the epithelial marker E‐cadherin in 549 and H125 cells treated with 20 µg/mL normoxic EVs or hypoxic EVs as detected by Western blot analysis, normalized to GAPDH. The experiment was repeated three times with independent samples. *P < .05 vs A549 and H12 cells upon PBS treatment. The data were measurement data and presented as mean ± standard deviation. Comparisons among multiple groups were analysed with the use of one‐way ANOVA, with a Tukey's*post hoc*test. Comparisons among multiple groups at different time‐points were analysed by repeated measures ANOVA, followed by Bonferroni*post hoc*test

A549 and H125 cells were adopted as the research objects in the current study. These cells were treated with the same concentration (20 µg/mL) of normoxic EVs or hypoxic EVs. We found that PKH67‐ (green fluorescence) labelled normoxic EVs or hypoxic EVs were internalized by A549 and H125 cells (Figure 2A). Next, we found that, compared with PBS treatment, the same concentration of hypoxic EVs promoted the proliferation, migration and invasion, and likewise the EMT of A549 and H125 cells (Figure 2B‐E).

**FIGURE 3:**
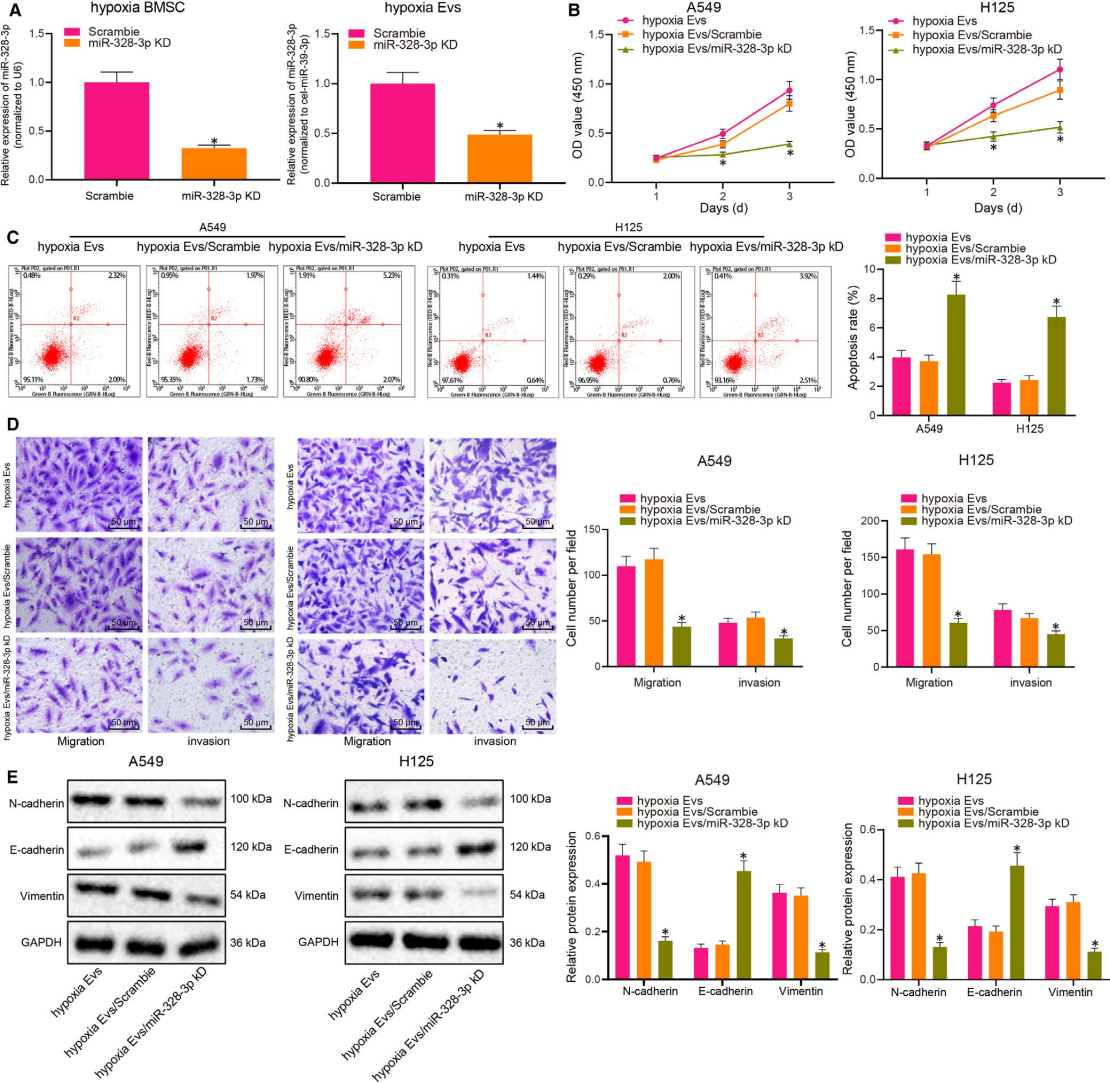
EV‐delivered miR‐328‐3p promotes the proliferation, migration, invasion and EMT of lung cancer cells in vitro. A, The expression of miR‐328‐3p in hypoxia‐treated BMSCs and EVs upon miR‐328‐3p knock‐down as determined by RT‐qPCR, normalized to cel‐miR‐39. B, The number of living cells at different time‐points as evaluated by CCK‐8 assay upon treatment with 20 µg/mL EVs from hypoxic BMSCs with miR‐328‐3p knock‐down. C, The apoptosis of A549 and H125 cells treated with 20 µg/mL EVs from hypoxic BMSCs with miR‐328‐3p knock‐down as detected by flow cytometry. D, The migration and invasion of A549 and H125 cells treated with 20 µg/mL EVs from hypoxic BMSCs with miR‐328‐3p knock‐down as detected by Transwell assay (scale bar = 50 µm). E, The content of the mesenchymal markers Vimentin, N‐cadherin and the epithelial marker E‐cadherin in 549 and H125 cells treated with 20 µg/mL EVs from hypoxic BMSCs with miR‐328‐3p knock‐down as detected by Western blot analysis, normalized to GAPDH. The experiment was repeated three times with independent samples. *P < .05 vs hypoxic EVs (A549 and H125 cells treated with EVs from BMSCs cultured under hypoxic conditions). The data were measurement data and presented as mean ± standard deviation. Data between two groups were compared using independent sample*t*test. Comparisons among multiple groups were analysed with the use of one‐way ANOVA, with Tukey's*post hoc*test. Comparisons among multiple groups at different time‐points were analysed by repeated measures ANOVA, followed by Bonferroni*post hoc*test

Subsequently, we down‐regulated the expression of miR‐328‐3p in hypoxia‐treated BMSCs using lentivirus. Relative to the scramble treatment group, miR‐328‐3p expression in hypoxia‐treated BMSCs and EVs was found to be decreased by 68% and 52%, respectively, after miR‐328‐3p knock‐down (Figure 3A). In addition, A549 cells and H125 cells were treated with EVs derived from hypoxia‐treated BMSCs with knock‐down of miR‐328‐3p. The results revealed that the proliferation, apoptosis, migration and invasion of A549 cells and H125 cells exhibited no significant changes between treatment with hypoxic EVs and scramble + hypoxic EVs, while the content of the mesenchymal markers (Vimentin and N‐cadherin) and the epithelial marker (E‐cadherin) were also unremarkable. However, the proliferation, migration and invasion of A549 cells and H125 cells were notably reduced, while apoptosis was augmented (Figure 3B‐D) in response to treatment with the hypoxic EVs containing miR‐328‐3p knock‐down. Moreover, the content of Vimentin and N‐cadherin was lowered, whereas that of E‐cadherin was elevated in A549 and H125 cells treated with hypoxic EVs containing miR‐328‐3p knock‐down (Figure 3E). Overall, these results demonstrated that hypoxic BMSC‐derived EVs containing miR‐328‐3p could induce the proliferation, migration, invasion and EMT of lung cancer cells.

### 3.3 Hypoxic BMSC‐derived EVs containing miR‐328‐3p induced in vivo tumour growth and metastasis

**FIGURE 4:**
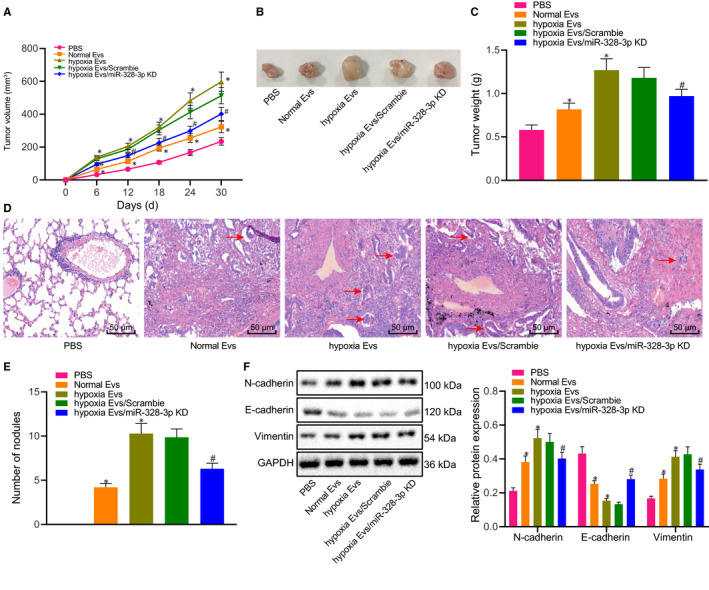
Hypoxic BMSC‐derived EVs containing miR‐328‐3p induce in vivo tumour growth and metastasis. BMSC‐derived EVs were resuspended in 100 µL sterile PBS and injected intravenously into the mice once every 2 d (100 µg each time) after 1 × 10^6^A549 cell injection. A, Tumour volume of mice injected with EVs from hypoxia‐treated BMSCs with miR‐328‐3p knock‐down after A549 cell injection monitored by a Vernier caliper. B, Representative images showing xenografts in nude mice injected with EVs from hypoxia‐treated BMSCs with miR‐328‐3p knock‐down after A549 cell injection. C, Tumour weight of mice injected with EVs from hypoxia‐treated BMSCs with miR‐328‐3p knock‐down on the 30th day of A549 cell injection. D, HE staining for metastatic tumour nodules in the lung of mice injected with EVs from hypoxia‐treated BMSCs with miR‐328‐3p knock‐down after A549 cell injection (scale bar = 50 µm; the arrow shows a metastatic tumour nodule). E, Statistical plot of metastatic tumour nodules in the lung of mice injected with EVs from hypoxia‐treated BMSCs with miR‐328‐3p knock‐down on the 30th day of A549 cell injection. F, The content of the mesenchymal markers Vimentin, N‐cadherin and the epithelial marker E‐cadherin in tissues of mice treated with EVs from hypoxia‐treated BMSCs with miR‐328‐3p knock‐down after A549 cell injection as detected by Western blot analysis, normalized to GAPDH. n = 10. The experiment was independently repeated three times. *P < .05 vs mice treated with PBS. #*P* < .05 vs hypoxic EVs/scramble treatment (mice injected with EVs from hypoxia‐treated BMSCs with scramble treatment after A549 cell injection). The data were measurement data and presented as mean ± standard deviation. Data between two groups were compared using independent sample*t*test. Comparisons among multiple groups were analysed with the use of one‐way ANOVA, with Tukey's*post hoc*test

Furthermore, the effects of hypoxic BMSC‐derived EVs containing miR‐328‐3p on tumour growth and metastasis were examined in vivo. A549 cells (1 × 10^6^) were injected into BALB/c nude mice via the tail vein, followed by injection with EVs cultured under normoxic or hypoxic conditions. Compared with PBS, EVs cultured under either normoxia or hypoxia conditions accelerated the tumour growth and increased the number of metastatic tumour nodules in the lung, with a more pronounced effect detected in the presence of hypoxic EVs. Relative to hypoxic exo or scramble treatment, hypoxic EVs containing miR‐328‐3p knock‐down led to decreased tumour growth and reduced number of metastatic tumour nodules in the lung (Figure 4A‐E). In addition, the results of Western blot analysis demonstrated that the contents of Vimentin and N‐cadherin in tumour tissues were increased, while that of E‐cadherin was decreased by EVs cultured under either normoxic or hypoxic conditions in contrast to PBS treatment; among the three treatment patterns, hypoxic EVs exerted the most substantial effects in terms of altering the content of EMT markers. Parallel to hypoxic EVs or scramble treatment, we found that hypoxic EVs containing miR‐328‐3p knock‐down brought about a marked decline in the contents of Vimentin and N‐cadherin, as well as an elevation in the E‐cadherin content (Figure 4F). Collectively, these results suggested that miR‐328‐3p delivered by hypoxic BMSC‐derived EVs was capable of promoting in vivo tumour growth and metastasis of lung cancer.

### 3.4 EV‐delivered miR‐328‐3p targeted the NF2 gene in lung cancer cells

**FIGURE 5:**
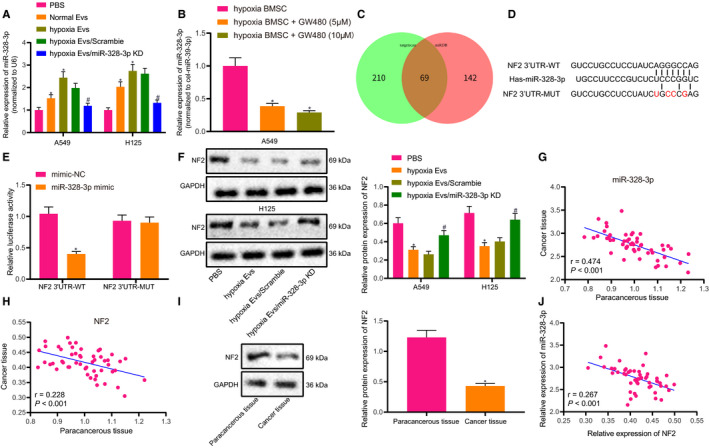
EV‐delivered miR‐328‐3p targets NF2 gene in lung cancer cells. A, The expression of miR‐328‐3p in A549 and H125 cells treated with 20 µg/mL hypoxic EVs as detected by RT‐qPCR, normalized to cel‐miR‐39. B, The expression of miR‐328‐3p in EVs of hypoxic BMSCs treated with GW480 detected by RT‐qPCR, normalized to cel‐miR‐39. C, Prediction of miR‐328‐3p downstream target genes by bioinformatics analysis. The two ellipses in the figure, respectively, represent the prediction results of the two databases, and the overlapping part represents the intersection of the two databases. D, The binding site of miR‐328‐3p to NF2 3′UTR predicted on the TargetScan website. E, The luciferase activity of NF‐WT and NF‐MUT in HEK293T cells transfected with miR‐328‐3p mimic as detected by dual‐luciferase reporter gene assay. F, The expression of NF2 in A549 and H125 cells treated with 20 µg/mL hypoxic BMSC‐derived EVs as detected by Western blot analysis. G, The expression of miR‐328‐3p in lung cancer and adjacent normal tissues as analysed by RT‐qPCR normalized to U6. H, The expression of NF2 in lung cancer and adjacent normal tissues as analysed by RT‐qPCR, normalized to β‐actin. I, The expression of NF2 in lung cancer and adjacent normal tissues as analysed by Western blot analysis, normalized to GAPDH. J, Correlation analysis between miR‐328‐3p expression and NF2 expression in lung cancer tissues. The experiment was repeated three times with independent sample. *P < .05 vs adjacent normal tissues, hypoxic BMSCs, mimic‐NC (HEK293T cells transfected with mimic‐NC) or PBS (A549 and H125 cells treated with PBS). #*P* < .05 vs hypoxic EVs/scramble treatment (A549 and H125 cells treated with EVs from hypoxia‐treated BMSCs with scramble treatment). The data were measurement data and presented as mean ± standard deviation. Data between two groups were compared using independent sample*t*test. Comparisons among multiple groups were analysed with the use of one‐way ANOVA, with Tukey's test*post hoc*test

The aforementioned results demonstrated that hypoxic BMSC‐derived EVs can be internalized by A549 and H125 cells. Subsequently, we explored whether hypoxic BMSC‐derived EVs exert this effect by delivering miR‐328‐3p. RT‐qPCR revealed that A549 and H125 cells presented with significantly increased expressions of miR‐328‐3p upon treatment with hypoxic EVs (Figure 5A). Meanwhile, the miR‐328‐3p expression was decreased after treatment of hypoxic BMSCs with the EV biogenesis/release inhibitor GW480 (Figure 5B). To unravel the underlying mechanism of carcinogenesis of BMSC‐derived EVs on cancer cells, the downstream target genes of miR‐328‐3p were predicted through various databases including miRDB, which indicated a total of 69 potential downstream target genes through the intersection of the prediction results (Figure 5C). Among the intersecting genes, NF2 in particular was suggested to possess a binding site for miR‐328‐3p, based on the prediction results of the TargetScan website (Figure 5D). In addition, dual‐luciferase reporter gene assay displayed that the luciferase activity of HEK293T cells in the presence of co‐transfection of miR‐328‐3p mimic and NF2 3′UTR‐WT was markedly decreased, while being unchanged upon other treatments (Figure 5E). As shown in Western blot analysis, A549 and H125 cells exhibited notably decreased NF2 expressions upon treatment with hypoxic BMSC‐derived EVs (Figure 5F). Additionally, the expression of miR‐328‐3p and NF2 was detected in lung cancer and adjacent normal tissues. The results revealed that relative to adjacent normal tissues, miR‐328‐3p was up‐regulated and NF2 was down‐regulated in lung cancer tissues, and their expressions had a negative correlation in lung cancer tissues (Figure 5G‐J). Overall, the aforementioned results demonstrated that EV‐delivered miR‐328‐3p targeted the NF2 gene to affect lung cancer cells.

### 3.5 miR‐328‐3p delivered by hypoxic BMSC‐derived EVs inhibited the Hippo pathway by targeting NF2

**FIGURE 6:**
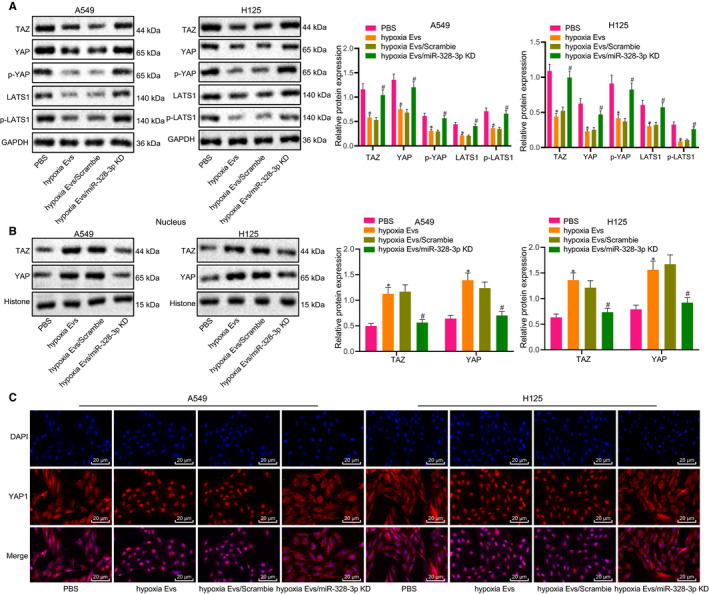
miR‐328‐3p delivered by hypoxic BMSC‐derived EVs inhibits the Hippo pathway by targeting NF2. A, The activation of the Hippo pathway in A549 and H125 cells treated with EVs from hypoxic BMSCs with miR‐328‐3p knock‐down as detected by Western blot analysis, normalized to GAPDH. B, The activation of the Hippo pathway in the nuclei of A549 and H125 cells treated with EVs from hypoxic BMSCs with miR‐328‐3p knock‐down as detected by Western blot analysis, normalized to GAPDH. C, Nuclear translocation of YAP and LATS1 in A549 and H125 cells treated with EVs from hypoxic BMSCs with miR‐328‐3p knock‐down as detected by immunofluorescence (scale bar = 20 µm). The experiment was repeated three times with independent samples. *P < .05 vs A549 and H125 cells treated with PBS. #*P* < .05 vs hypoxic EVs/scramble treatment (A549 and H125 cells treated with EVs from hypoxia‐treated BMSCs with scramble treatment). The data were measurement data and presented as mean ± standard deviation. Comparisons among multiple groups were analysed with the use of one‐way ANOVA, with Tukey's*post hoc*test

In addition, we set about to confirm our speculation that miR‐328‐3p delivered by hypoxic BMSC‐derived EVs could inhibit the activation of the Hippo pathway by targeting the NF2 gene. Initially, the activation of the Hippo pathway was detected in A549 and H125 cells treated with miR‐328‐3p delivered by hypoxic BMSC‐derived EVs. Compared with PBS, hypoxic EVs were found to notably reduce the extent of YAP and LATS1 phosphorylation and the expression of TAZ, but to elevate the expression of YAP and TAZ in the nuclei of A549 and H125 cells. Relative to hypoxic EVs or scramble treatment, hypoxic EVs containing miR‐328‐3p knock‐down showed an increase in the extent of YAP and LATS1 phosphorylation and the expression of TAZ, but decreased the expressions of YAP and TAZ in the nuclei of A549 and H125 cells (Figure 6A‐C). The above results demonstrated that miR‐328‐3p delivered by hypoxic BMSC‐derived EVs could inactivate the Hippo pathway via NF2.

### 3.6 Circulating miR‐328‐3p delivered by EVs was bioactive in lung cancer patients

**FIGURE 7:**
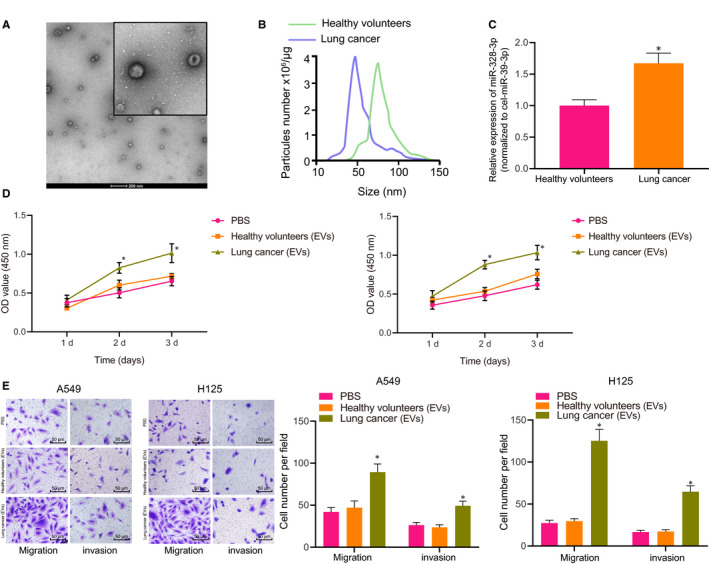
Circulating miR‐328‐3p delivered by EVs is bioactive in lung cancer patients. A, Morphological characteristics of EVs isolated from the serum of lung cancer patients observed under a scanning electron microscope (scale bar = 200 nm). B, NTA analysis of the EVs isolated from the serum of lung cancer patients. C, The expression of miR‐328‐3p in the EVs from the serum of lung cancer patients and healthy individuals as detected by RT‐qPCR, normalized to cel‐miR‐39. D, The viability of A549 cells and H125 cells after co‐culture with EVs from the serum of lung cancer patients and healthy individuals as detected by CCK‐8 assay. E, The migration and invasion of A549 cells and H125 cells after co‐culture with EVs from the serum of lung cancer patients and healthy individuals as detected by Transwell assay (scale bar = 50 µm). *P < .05 vs A549 cells and H125 cells treated with EVs from the serum of healthy individuals or treated with PBS. The data were measurement data and presented as mean ± standard deviation. Data between two groups were compared using independent sample*t*test. Comparisons among multiple groups were analysed with one‐way ANOVA and Tukey's*post hoc*test

To study the potential clinical significance of circulating miR‐328‐3p delivered by EVs in lung cancer patients, EVs were randomly separated and identified in the serum samples of 5 lung cancer patients and 5 healthy individuals. The purified EVs were 50‐200 nm vesicles (Figure 7A, B) as measured by NTA and electron microscopy. There were no marked differences in the concentration of EVs between lung cancer patients and healthy individuals. RT‐qPCR results illustrated that the expression of miR‐328‐3p in the EVs was significantly higher in lung cancer patients compared to the healthy individuals (Figure 7C). To explore further whether circulating EVs could regulate the progression of lung cancer, A549 and H125 cells were treated with EVs isolated from serum samples of lung cancer patients. Based on the results, relative to PBS, EVs from lung cancer patients significantly increased the viability, migration and invasion of A549 cells and H125 cells (Figure 7D, E). Collectively, these findings demonstrated that EVs were bioactive in lung cancer patients.

## 4 DISCUSSION

Lung cancer is the leading cause of cancer‐related deaths across the world, imposing a huge burden on healthcare systems in every country.^25^ An increasing number of researchers have highlighted the importance of EVs in several lung diseases.^26^ In the current study, we aimed to investigate whether EV‐delivered miR‐328‐3p derived from hypoxic BMSCs influences the occurrence and progression of lung cancer and to uncover evidence demonstrating its novel tumour promoting role.

Initially, we discovered that miR‐328‐3p was up‐regulated in lung cancer tissues and EVs derived from hypoxic BMSCs. miR‐328 was previously detected to be an up‐regulated gene in lung adenocarcinomas.^27^ Another study demonstrated that the expression of miR‐328 was notably higher in patients with non‐small cell lung cancer than in healthy individuals.^14^ Moreover, M2 macrophage‐derived exosomal miR‐328 has also been shown to promote the development of rat pulmonary fibrosis through FAM13A.^28^ Meanwhile, hypoxia is recognized as a typical feature of locally advanced solid tumours, which further accelerates tumour development.^29^ Similar to our present findings, a previous study found that exosome production was augmented in lung cancer cells under hypoxic conditions, and that another miRNA, miR‐23a, exhibited significantly increased expression in the hypoxia‐induced exosomes.^30^ Moreover, miR‐24 showed a higher expression in BMSC‐derived exosomes cultured under hypoxic conditions compared to normoxic conditions.^31^

Another aspect of the current study was our finding that EV‐delivered miR‐328‐3p derived from hypoxic BMSCs promoted the proliferation, invasion and migration of lung cancer cells, while inhibiting their apoptosis; in vivo assay results further confirmed that the EV‐delivered miR‐328‐3p accelerated tumour growth. In further support of this result, another study reported that miR‐328 is responsible for the brain metastasis of non‐small cell lung cancer and regulates its migration.^32^ Also in line with our findings, down‐regulated miR‐328 was previously found to inhibit tumour growth and promote cell apoptosis of lung cancer in vivo.^15^ Moreover, EV‐delivered miRs have also been recently highlighted to exert important functions in general tumour cell biology. For instance, EVs containing miR‐200 were reported to facilitate the metastasis of breast cancer cells.^33^ Human umbilical cord mesenchymal stem cell‐derived EVs, by delivering miR‐410, have also been previously demonstrated to accelerate the development of lung adenocarcinoma.^34^ Meanwhile, human BMSC‐derived exosomes have also been discovered to be capable of accelerating in vivo tumour growth.^35^ Similar to our results, BMSC‐derived exosomal miR‐208a could facilitate the proliferation, migration and invasion of osteosarcoma cells.^36^ Taken together, aforementioned literature results support our present discovery that miR‐328‐3p promotes the development of lung cancer both in vitro and in vivo through the delivery of EVs derived from hypoxic BMSCs.

Another key finding of the current study was that NF2 was proved to be the target gene of miR‐328‐3p. Furthermore, we found that, by targeting NF2, which was down‐regulated in lung cancer tissues, EV‐delivered miR‐328‐3p down‐regulated the Hippo pathway to influence the progression of lung cancer. The Hippo pathway is well‐known to be implicated in the biological functions of a variety of cancers.^37^ In concord with our present findings, the activated Hippo pathway was previously shown to be capable of inhibiting the proliferation and invasion of lung cancer cells.^38^ Also, the NF2 gene is reported to exert its effects through the Hippo pathway to regulate cell proliferation and apoptosis.^39^ Consistent with this scenario, NF2 was detected to be down‐regulated in lung cancer cells, which aided in accelerating the proliferation and migration of lung cancer cells.^18^ In the current study, online prediction, dual‐luciferase reporter gene assay, RT‐qPCR and Western blot analysis, as well as Pearson's correlation analysis, all concurred in showing the targeting relationship between miR‐328‐3p and NF2. In addition to this, we documented bioactivity of circulating miR‐328‐3p in patients with lung cancer. In fact, circulating miRs have been highlighted previously to have potential as critical biomarkers for prognosis of lung cancer.^40^ Interestingly, recent research has highlighted the role of BMSC‐derived exosomes in the promotion of cell proliferation and survival, which is achieved via delivery of multiple bioactive molecules, including miRs.^41^

**FIGURE 8:**
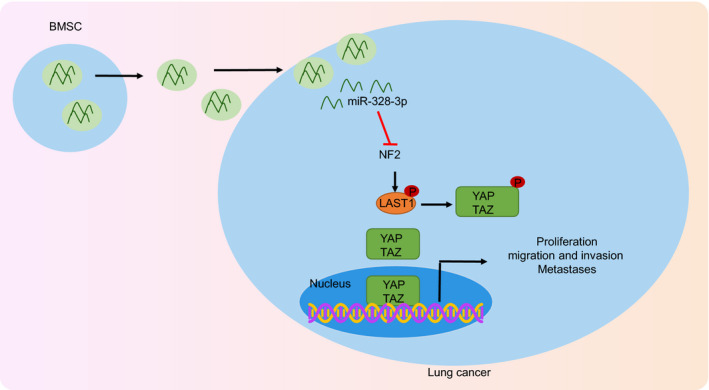
The proposed regulatory mechanism illustrating the role of hypoxic BMSC‐derived EVs containing miR‐328‐3p in lung cancer. miR‐328‐3p can be delivered from BMSCs to lung cancer cells by hypoxic BMSC‐derived EVs, thereby targeting NF2 in lung cancer cells. The down‐regulation of NF2 leads to the inhibition of the Hippo pathway, which promotes the proliferation, migration, invasion and EMT of lung cancer cells

In summary, we demonstrated that hypoxic BMSC‐derived EVs could deliver miR‐328‐3p to target the NF2 gene, which inhibited the Hippo pathway, thereby promoting the occurrence and progression of lung cancer (Figure 8). Our findings highlight that EV‐delivered miR‐328‐3p is a promising target for the treatment of lung cancer. However, the feasibility of such an approach naturally requires confirmation in large‐scale clinical trials to improve the outcomes of patients plagued by lung cancer.

## CONFLICT OF INTEREST

The authors declare that there is no conflict of interest.

## AUTHOR CONTRIBUTIONS

**Xi Liu:** Conceptualization (equal); Funding acquisition (equal); Writing‐original draft (equal). **Feng Jiang:** Data curation (equal); Resources (equal). **Zhiliang Wang:** Formal analysis (equal); Methodology (equal). **Lang Tang:** Project administration (equal); Validation (equal). **Bin Zou:** Software (equal); Visualization (equal). **Pengfei Xu:** Conceptualization (equal); Supervision (equal). **Tenghua Yu:** Funding acquisition (equal); Investigation (equal); Writing‐review & editing (equal).

## Data Availability

Research data not shared.
