# Corrigendum

**DOI:** 10.1111/jcmm.17193

**Published:** 2022-04-05

In Tenghua Yu et al., the ‘Nanosight particle tracking analysis of the EVs isolated from BMSCs cultured under normoxic and hypoxic conditions’ in Figure 1B, the original result analysis curve result was inaccurate. The correct figure is shown below. The authors confirm all results and conclusions of this article remain unchanged.

**FIGURE 1:**
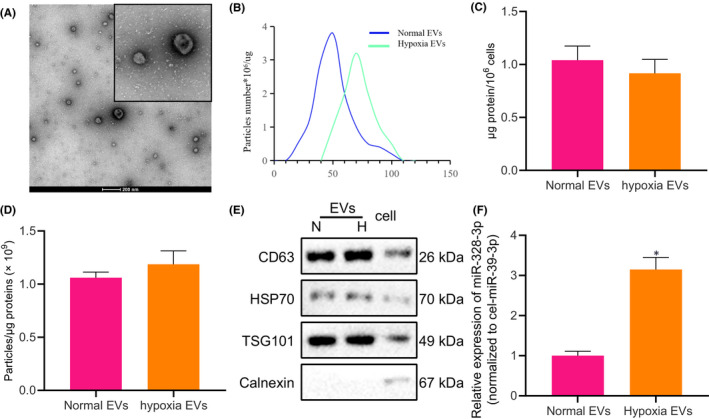
miR‐328‐3p is up‐regulated in hypoxic BMSC‐derived EVs. (A) Morphological characteristics of EVs isolated from BMSCs observed under a scanning electron microscopy (scale bar = 200 nm). (B) Nanosight particle tracking analysis of the EVs isolated from BMSCs cultured under normoxic and hypoxic conditions. (C) Quantification of equivalent protein EVs produced by 106 MSCs by BCA analysis. (D) Correlation of the quantity of EVs with the amount of protein. (E) The presence of CD63, TSG101 and HSP70 and the absence of calnexin in BMSC‐derived EVs, as detected by Western blot analysis, normalized to GAPDH. (F) Relative expression of miR‐328‐3p determined by RT‐qPCR in EVs isolated from BMSCs cultured under normoxic and hypoxic conditions, normalized to cel‐miR‐39, showing the increase of miR‐328‐3p in EVs from BMSCs cultured under hypoxic conditions. In this examination, the hypoxic extracellular vesicles were isolated from the supernatant of BMSCs cultured under hypoxic conditions, while normal extracellular vesicles were isolated from the supernatant of BMSCs cultured under normal conditions. ‘EVs’ is the abbreviation of extracellular vesicles. The experiment was repeated three times with independent samples. *p < 0.05 vs the EVs from BMSCs cultured under normoxic conditions. The data were measurement data and presented as mean ± standard deviation. Data between two groups were compared using independent sample t‐test
